# Comprehensive Analysis Identifies and Validates the Tumor Microenvironment Subtypes to Predict Anti-Tumor Therapy Efficacy in Hepatocellular Carcinoma

**DOI:** 10.3389/fimmu.2022.838374

**Published:** 2022-07-18

**Authors:** Haohan Zhang, Yi Yao, Jie Wu, Jin Zhou, Chen Zhao, Junju He, Bin Xu

**Affiliations:** ^1^ Cancer Center, Renmin Hospital of Wuhan University, Wuhan, China; ^2^ Hubei Provincial Research Center for Precision Medicine of Cancer, Wuhan, China

**Keywords:** hepatocellular carcinoma, tumor immunology, tumor microenvironment subtypes, precise treatment, immunotherapy, tumor-infiltrating lymphocytes, tumor stromal cells, stem cells

## Abstract

**Objective:**

The objective of this study was to explore and verify the subtypes in hepatocellular carcinoma based on the immune (lymphocyte and myeloid cells), stem, and stromal cells in the tumor microenvironment and analyze the biological characteristics and potential relevance of each cluster.

**Methods:**

We used the xCell algorithm to calculate cell scores and got subtypes by k-means clustering. In the external validation sets, we verified the conclusion stability by a neural network model. Simultaneously, we speculated the inner connection between clusters by pseudotime trajectory analysis and confirmed it by pathway enrichment, TMB, CNV, etc., analysis.

**Result:**

According to the results of the consensus cluster, we chose k = 4 as the optimal value and got four different subtypes (C1, C2, C3, and C4) with different biological characteristics based on infiltrating levels of 48 cells in TME. In univariable Cox regression, the hazard ratio (HR) value of C3 versus C1 was 2.881 (95% CI: 1.572–5.279); in multivariable Cox regression, we corrected the age and TNM stage, and the HR value of C3 versus C1 was 2.510 (95% CI: 1.339–4.706). C1 and C2 belonged to the immune-active type, C3 and C4 related to the immune-insensitive type and the potential conversion relationships between clusters. We established a neural network model, and the area under the curves of the neural network model was 0.949 in the testing cohort; the same survival results were also observed in the external validation set. We compared the differences in cell infiltration, immune function, pathway enrichment, TMB, and CNV of four clusters and speculated that C1 and C2 were more likely to benefit from immunotherapy and C3 may benefit from FGF inhibitors.

**Discussion:**

Our analysis provides a new approach for the identification of four tumor microenvironment clusters in patients with liver cancer and identifies the biological differences and predicts the immunotherapy efficacy between the four subtypes.

## Introduction

Hepatocellular carcinoma (HCC) is one of the most lethal malignancies worldwide, with low survival rates in advanced-stage patients and minimal improvement in survival trends. The tumor microenvironment (TME) consists of many cell types, including immune infiltrates (lymphocyte and myeloid cells), cancer-associated fibroblasts (CAFs), and vascular endothelial cells. Immune cells and stromal cells, which are two major types of non-tumor cell components, play crucial roles in tumor progression and metastasis (1, 2). Previous studies have been conducted on the relationship between tumor-infiltrating immune cells and clinical outcomes (3). However, other components in the TME, such as stromal cells, also affect the therapeutic outcome (4), and the research on the comprehensive compendium of the cell landscape in TME is still lacking in LIHC.

Immunotherapy has received tremendous attention and is revolutionizing cancer treatment. Immune checkpoint inhibitors (ICIs) can reverse the immunosuppressive microenvironment by decreasing the potential of tumor immune escape, resulting in a noteworthy improvement of prognosis (5). In TME, multiple factors affect immunotherapy. The stem cells and stromal cells could suppress pro-inflammatory processes and promote the immune tolerance (6). Accordingly, not all cancer patients exhibit the same response to immunotherapy, and it is of utmost importance to identify the immunodominant population to help clinicians conduct immunotherapy or immunotherapy-based combination strategies.

In view of this situation, this study aimed at establishing the tumor microenvironment subtypes based on 48 types of cells, including immune (lymphocyte and myeloid cells), stem, and stromal cells in TME. We investigated the differences in biological characteristics among different subtypes, including infiltration of immune cells, tumor microenvironment status, tumor mutations, and the differences in prognosis and the efficacy of immunotherapy, which may refer to current research on treatment strategies for patients with LIHC.

## Methods

### Data Source

RNA-seq data of LIHC patients were downloaded from The Cancer Genome Atlas (TCGA) (https://portal.gdc.cancer.gov/) and further normalized into transcripts per kilobase (TPM) for analysis. Normalized microarray gene expression data of the Hoshida Y et al. cohort (GSE10141) were available from the Gene Expression Omnibus (GEO) database (https://www.ncbi.nlm.nih.gov/geo/), and LIHC transcriptome and clinical data of the Japanese cohort were available from the International Cancer Genome Consortium (ICGC) database (https://dcc.icgc.org). TCGA dataset was used to investigate the immune subtypes of tumor microenvironment subtypes, and the Hoshida Y et al. cohort and Japanese cohort were independently used for external validation.

The enrichment scores of 64 cells in TME were inferred by the xCell algorithm, which integrated the advantages of gene-set enrichment with deconvolution approaches to remove dependencies between cell types (7). When selecting cell types, we first removed the other cell type family, which mainly included neural and sebaceous cells, in the algorithm. According to the cell p-value and the standard deviation of cell scores, we removed 8 cell types again. The tumor purity of TCGA patients was inferred by ESTIMATE (Estimation of Stromal and Immune cells in Malignant Tumors using Expression data), ABSOLUTE, LUMP (leukocyte unmethylation for purity), and CPE (consensus measurement of purity estimations) algorithms, and the immune subtypes of TCGA patients were provided in the study of Vésteinn Thorsson et al. (8). Moreover, related features, including the signature scores of tumor proliferation, wound healing, macrophage regulation, lymphocyte infiltration signature, IFN-γ response and TGF-β response, leukocyte fraction, tumor-infiltrating lymphocyte (TIL) regional fraction, and intratumor heterogeneity, were also used in our study.

### Non-Supervisor Clustering and Identification of TME Subtypes

We performed the k-mean consensus cluster method to identify tumor microenvironment subtypes for TCGA patients, based on the R package “ConsensusClusterPlus”. Performance of consensus matrix, empirical cumulative distribution function (CDF) plots, and relative change in area under CDF curve were considered when selecting optimal k value. The tumor microenvironment subtypes of patients in the Japanese cohort and the Hoshida Y et al. cohort were determined by a neural network model, which was trained and internally validated in TCGA dataset (the training and testing cohorts were randomly divided at a ratio of 7:3).

The neural network consisted of the input layer, hidden layers, and output layer. We set the cell matrix as the input layer and the subtype result as the output layer. The setting of the hidden layer neural nodes refers to the following formula:


$${\text{N}}_{h}=\frac{N_{s}}{a*\left(N_{i}+N_{o}\right)}$$

*N_i_* is the number of neurons in the input layer; *N_o_* is the number of neurons in the output layer; *N_s_* is the number of training set samples; and *a* could be an arbitrary variable.

We used the receiver operating characteristic (ROC) curve analysis to confirm the performance of the prognostic model.

### Survival Analysis

The KM and Cox regression analyses were used to calculate the significance of differences in the overall survival (OS) for categorical variables. The statistical difference of the OS in the KM curve analysis was compared using the log-rank test, and the pairwise comparison was performed between multiple groups. For continuous variables, Cox regression was used to calculate the hazard ratio (HR) and significance of differences in the OS.

### Gene Set Enrichment Analysis

R package “DESeq2” implement procedures were utilized for the differential expression analysis between any cluster and other patients (9). The genes with a false discovery rate (FDR, also known as Benjamini–Hochberg-adjusted p-values) < 0.05 and absolute log-transformed fold change (log2FC) > 1.0 were defined as differential expression genes. The gene list is then ranked by log2FC and studied using gene set enrichment analysis, which was computed by the R package “ClusterProfiler” (10). The signaling pathway information in the Molecular Signatures Database (MSigDB) (https://www.gsea-msigdb.org/gsea/msigdb/) was used. The pathway enrichment scores for each patient were calculated by the gene set variation analysis (GSVA).

### Genomic Mutation and Copy Number Variants

The gene mutation data of LIHC were downloaded from TCGA database. The “Maftools” R package was applied to visualize the gene mutations and type of the mutation (11). The copy number data were recognized by GISTIC 2.0 (12).

### Pseudotime Trajectory Analysis

Through inversed pseudotime trajectory analysis, we reduced the dimensions of all samples on the same plane, observed the distribution between different clusters, and explored the latent associations between clusters. Through the pseudotime ordering of each patient, we attempted to reveal the patient’s possible disease development directions (13).

### Evaluation of Drug Sensitivity and Patients’ Response to Immunotherapy

Using the pRRophetic algorithm (14), a ridge regression model was established to predict the sensitivity value (IC50) of 51 drugs for LIHC patients of TCGA, the Japanese cohort, and the Hoshida Y et al. cohort based on the expression profile. pRRophetic is a popular enrichment algorithm, which was extensively utilized in medical studies (15–19). The potential response of patients to immunotherapy was inferred by the tumor immune dysfunction and exclusion (TIDE) score, which calculates how the expression of each gene in the tumor interacts with the level of cytotoxic T-cell infiltration to affect patient survival (20). Generally, a lower TIDE score predicts a better response to immunotherapy.

### Other Statistical Analysis

Immune and stromal scores were calculated using the ESTIMATE algorithm, which was provided in the R package “estimate,” and the correlation analysis was conducted based on the Spearman method. The Kruskal–Wallis test examined the statistical difference of distribution in three or more groups, and the Wilcoxon test compared that of two groups. The missing value of clinical data in our study was imputed by multiple imputation methods based on chain equitation. The FDR was calculated by the Benjamin–Hochberg method for adjusting the p-value in multiple comparisons.

## Results

### The Association Between 48 Cells in TME and Clinical Characteristics in TCGA Patients

**Figure 1:**
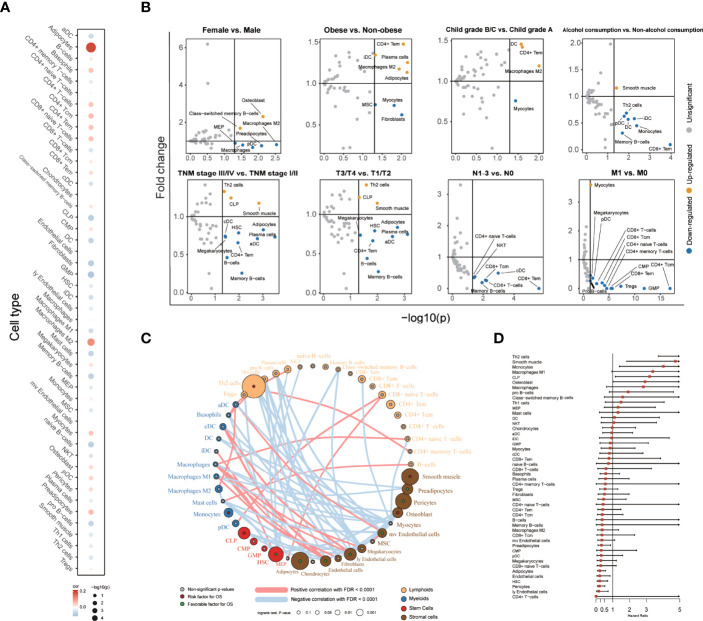
The association between 48 cells in TME and clinical characteristics with patients. **(A)** Correlation between age and cell score. The size of each cell represented the correlation between age and cell score. Spearman correlation coefficients and the associated p-value (Spearman) were shown. **(B)** Volcano plot diagrams showed the comparison of cell infiltration levels. The significance (p-value) versus and fold change were plotted on the X-axis and T-axis. **(C)** Landscape of the TME in LIHC. Cellular interaction of the TME cell types. The lymphoid cells are marked by yellow; the myeloid cells are marked by blue; stem cells are marked by red; and stromal cells are marked by brown. The size of each cell represents the survival impact of each TME cell type, which was calculated by log10 (log-rank test p values indicated). Risk factors are indicated in red, and favorable factors for overall survival are indicated in green. The lines connecting TME cells represent cellular interactions. The thickness of the line represents the strength of correlation estimated by Spearman correlation analysis. Positive correlation is indicated in red and negative correlation in blue. **(D)** Forest plots showing multivariable Cox regression analyses of the cell score, and each score is adjusted by age and TNM stage.

Based on the xCell algorithm, a total of 48 types of cells, including immune (including lymphocyte and myeloid cells), stem, and stromal cells in TME, were available for analysis in LIHC. The correlation between tumor microenvironment cells and the age of patients was calculated (**Figure 1A**). The adipocytes and macrophages M2 were significantly positively correlated with age. In contrast, the granulocyte-macrophage progenitor was negatively correlated with age. Although CD8+ T cells, CD4+ memory T cells, CD4+ naïve T cells, T helper 1 (Th1) cells, and T helper 2 (Th2) cells were positively associated with the patients’ age, the associations were insignificant. The associations between age and each cell score in the TME of patients are shown in the **Supplementary Figure 1**.

Subsequently, we compared the estimated cell scores across different genders, levels of obesity, Child–Pugh grade, alcohol consumption, TNM stages, T stages, N stages, and M stages (**Figure 1B**). In comparing different levels of obesity and different Child–Pugh grades, the CD4+ T effector memory cells, immature dendritic cells, and macrophages M2 had higher scores in obese people and in the Child–Pugh grade B/C population. Maturation of dendritic cells could act as antigen-presenting cells and initiate the host anticancer immune response (21), but patients with obesity and poor liver function grading may have maturation disturbance of dendritic cells. When comparing the cell scores between TNM stages, the score of conventional dendritic cells and activated dendritic cells decreased in stage III/IV and stage T3/T4, which could activate cytotoxic T lymphocytes cross-presenting antigens (22).

We utilized the TME cell network to depict the comprehensive landscape of tumor–immune cell interactions, cell lineages, and their effects on the overall survival of patients with LIHC (**Figure 1C**, **Supplemental Table 1**). Through univariable Cox analysis, we found that only the Th 2 cell score was associated with poor prognosis in lymphoid, which was consistent with previous research conclusions (23). Many cells, such as adipocytes and endothelial cells, were associated with favorable prognosis, which was usually thought to accelerate tumor metastasis (24, 25). We also calculated the HR of these cells using multivariable Cox analysis to correct the age and TNM stage (**Figure 1D**). Similar to the result of univariable Cox analysis, we found that Th 2 cells were associated with poor prognosis, and endothelial cells, etc., were associated with favorable prognosis.

### Identification of Subtypes in TME of Patients With LIHC

**Figure 2:**
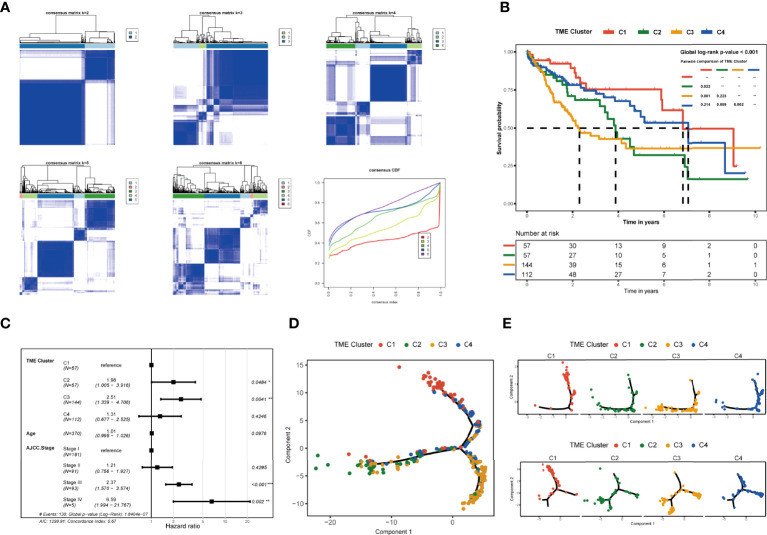
Identification and clinical characteristics of TME subgroups. **(A)** Consensus clustering displaying the robustness of sample classification using multiple iterations (×1,000) of k-means clustering. The consensus distribution function (CDF) depicting the cumulative distribution from consensus matrices at a given cluster number (k). **(B)** Kaplan–Meier curves for overall survival of 370 patients in TCGA database showed the association between TME subtypes and overall survival (global log-rank test, p = 0.00033). **(C)** Forest plots showing multivariate Cox regression analyses of the TME class, age, and TNM stage on the overall survival of LIHC patients. **(D)** Pseudotime trajectory analysis speculated the developmental relationship of the clusters based on the differential genes. **(E)** Pseudotime trajectory analysis of 370 patients in TCGA based on the glycan biosynthesis and metabolism pathway-related genes and cellular processes (including transport and catabolism, cell growth and death, cellular community, and cell motility) pathway-related genes. *p≤ 0.05, **p≤ 0.01, ***p≤ 0.001.

The consensus matrix was used as the similarity matrix to define the final clusters. Sample classification robustness was analyzed by consensus clustering, which involved k-means clustering by resampling randomly selected tumor profiles (**Figure 2A**). According to the results of the consensus cluster and the areas under the curve of the consensus distribution function (CDF) plot, we chose k = 4 as the optimal value and divided 370 LIHC patients into four different subtypes (C1, C2, C3, and C4) with 57 samples in C1, 57 samples in C2, 144 samples in C3, and 112 samples in C4. In parallel, the overall survival in the TME clusters was significantly different (p of log-rank test = 0.0026, **Figure 2B**). Compared to patients of C1, those of C2 and C3 had a significantly poorer overall survival. In univariable Cox regression, the HR value of C2 versus C1 was 2.128 (95% CI: 1.087–4.165), that of C3 versus C1 was 2.881 (95% CI: 1.572–5.279), and that of C4 versus C1 was 1.413 (95% CI: 0.740–2.698). In multivariable Cox regression, the HR value of C2, C3, or C4 versus C1 was 1.983 (95% CI: 1.005–3.916), 2.510 (95% CI: 1.339–4.706), or 1.307 (95% CI: 0.677–2.525), respectively, by correcting the age and TNM stage (**Figure 2C**).

To explore the potential relationship between the four tumor microenvironment clusters, we used pseudotime ordering to analyze the development of the four subtypes (**Figure 2D**). According to the result, the patients in C1 and C3 were seated at both ends of the pseudotime ordering analysis, while the patients of C2 and C4 were in the middle of pseudotime ordering. The result of the pseudotime ordering analysis was similar to the overall survival result of each cluster. The pseudotime ordering suggested that C1 and C4 may be at a similar stage in the glycan biosynthesis and metabolism pathways and cellular process pathways (including transport and catabolism, cell growth and death, cellular community, and cell motility) and have a potential timing relationship with C2 and C3 (**Figure 2E**, **Supplementary Figure 2**). In carbohydrate metabolism-, energy metabolism-, and nucleotide metabolism-related pathways and all cellular process-related pathways, we observed that C2 and C4 were on different timing stages, which may have a potential temporal evolution relationship (**Supplementary Figure 2**). We discussed the more particular biological characteristics and enrichment analysis in the next.

### The Immune Characteristics in Different TME Clusters

**Figure 3:**
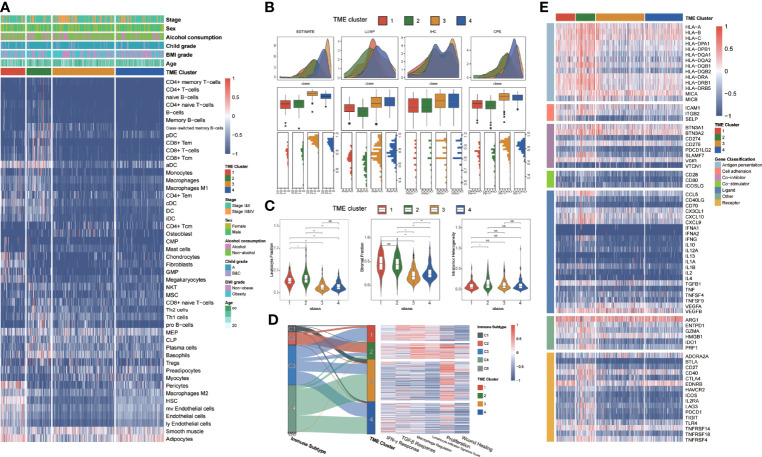
The immune characteristics of different TME clusters. **(A)** Unsupervised clustering of TME cells for 370 patients in TCGA database. Clinical stage, sex, alcohol consumption, Child grade, BMI grade, age, and TME cluster were shown as patient annotations. **(B)** The boxplots showed the comparison of tumor purities with ESTIMATE, LUMP, IHC, and CPE, respectively. The thick line represents the median value. The bottom and top of the boxes were the 25th and 75th percentiles (interquartile range). The whiskers encompassed 1.5 times the interquartile range. The statistical difference of four groups was compared through the Kruskal–Wallis test. **(C)** Violin plots showed the comparison of immunocompetence with leukocyte fraction, stromal fraction, and intratumor heterogeneity respectively. The differences between every two groups were compared through the Kruskal–Wallis test. **(D)** Sankey diagram illustrates change between the immune subtype and TME cluster in each patient. Heatmap of IFN-γ response, TGF-β response, macrophage regulation, lymphocyte infiltration, proliferation, and wound healing in different TME clusters. **(E)** Heatmap of immune genes that were differentially expressed in patients from different TME clusters. *p≤ 0.05, **p≤ 0.01, ns: p> 0.05.

To further characterize and understand the biological and immune differences and connections among these TME clusters, we contrasted the difference of cell scores in each TME cluster. As illustrated in **Figure 3A**, we observed a higher CD8+ T cell infiltration, including that of the CD8+ T cells, CD8+ T effector memory cells, and CD8+ T center memory cells, in C2 and C1 patients. Meanwhile, macrophages M1 and dendritic cells had higher cell scores in patients of C1 and C2. Concurrently, the other two clusters (C3 and C4) showed lower infiltration levels of CD8+ T cells, and the patients of C3 had lower endothelial cell scores.

The ESTIMATE, LUMP, IHC, and CPE scores from each TME cluster were also compared, which provided a qualitative estimation of tumor purity (**Figure 3B**). We observed that patients of C3 had higher tumor purity than other clusters. We counted the leukocyte fraction, stromal fraction, and intratumor heterogeneity in each subtype (**Figure 3C**). Notably, there was no significant difference in intratumor heterogeneity among the four subgroups, but C3 had the lowest leukocyte fraction and stromal fraction scores.

By probing the association between six identified immune subtypes and TME clusters, we found that most wound healing immune subtype patients belonged to TME C3, which means a high proliferation rate and an association with worse survival (**Figure 3D**). In contrast to wound healing, the IFN-γ-dominant subtype principally flowed to TME C2, which usually had the highest macrophages M1 and CD8+T cells. The lymphocyte-depleted subtype had minimal T helper cells and mainly flowed to TME C3 and C4. Furthermore, we discovered that C1 and C2 had higher lymphocyte infiltration and macrophage regulation scores than the other clusters, and C2 had higher macrophage regulation than others (**Figure 3E**). Simultaneously, C1 and C4 had a lower proliferation rate than C2 and C3, and C3 had the lowest IFN-γ signature. To seek the difference of immunocompetence between each cluster, we examined the immunomodulatory gene expression in each cluster (**Figure 3D**). Almost all antigen presentation genes were highly expressed in C2 and C1, and the receptor genes had a high gene expression in C2.

### The Analyses of Differentially Expressed Genes and Enriched Functions Between Different TME Clusters

**Figure 4:**
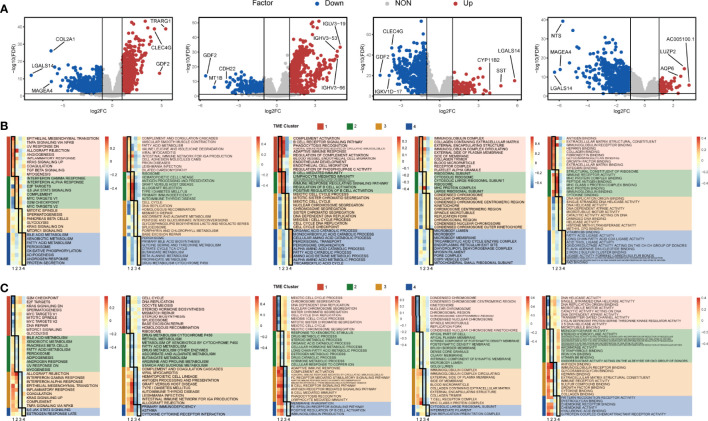
Differentially expressed genes and enriched functions in the TME cluster. **(A)** The volcano map showed the differentially expressed genes of each cluster. Red dots represent upregulated genes, blue dots represent downregulated genes, and gray dots represent no differentially expressed genes. **(B)** The Hallmark, KEGG, Gene Ontology Biological Process (GOBP), Gene Ontology Cellular Component (GOCC), and Gene Ontology Molecular Function (GOMF) enrichment analyses were performed for upregulated genes in each cluster, respectively. **(C)** The above enrichment analyses were performed for downregulated genes in each cluster, respectively.

We selected the top quarter with the most considerable variance from all genes to analyze the differential expression of genes in each TME cluster (**Figure 4A**, **Supplemental Table 2**). The highly altered genes involved in different TME clusters were TRARG1, CLEC4G, and GDF2 in C1; immunoglobulin lambda variable cluster family genes in C2; LGALS14, SST, and CYP11B2 in C3; and Lnc-NPVF-2, LUZP2, AQP6 in C4.

Then we used the gene sets in the MSigDB database to annotate the enriched biological functions, selected the significant enrichment pathway, and scored each patient by GSVA (**Figure 4B**). In TME C1, immune response-related pathways were highly enriched, including TGF-β signaling, complement activation, and B-cell receptor signaling pathway. The pathway enrichment of TME C2 was similar to that of C1, and those immune-related pathways were also enriched in C2. Nevertheless, IFN-α and IFN-γ response pathways and MYC target pathways were highly enriched in C2, specifically. Meanwhile, the cell cycle-related pathways were activated in TME C3, and the metabolism and decomposition-related pathways were activated in TME C4. In contrast, we found that cell cycle and cell proliferation-related pathways were the least enriched in C1, meaning that TME C1 may have the lowest cell proliferation rate. The metabolism-related and the oxidation–reduction (redox) reaction-related pathways were lowly enriched in C2 (**Figure 4C**). The downregulated pathways of C3 and C4 were highly similar, but C1 and C3 were opposite, especially in immune-related pathways (**Figures 4B, C**). Although the enrichment of immune pathways was similar in C1 and C2, the DNA replication and cell-cycle pathways were more enriched in C2. The same pattern also appeared in C3 versus C4, the metabolic-related pathways of C4 were more enriched, and the cell proliferation-related pathways were more enriched in C3 (**Supplementary Figure 3**).

### Genomic Alteration Landscape of Different TME Clusters

**Figure 5:**
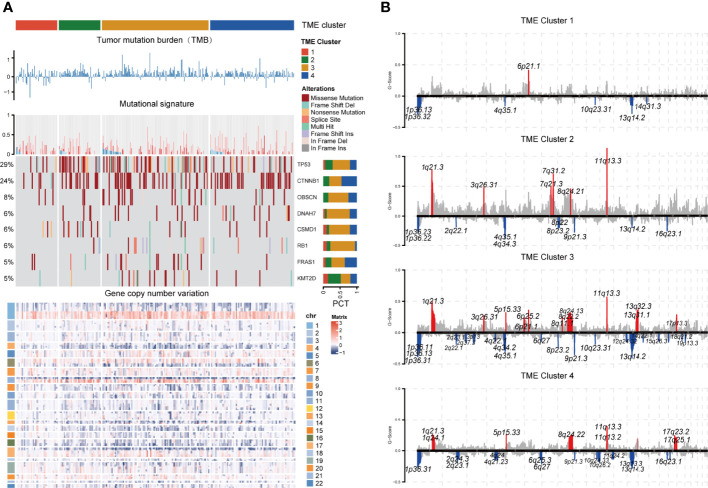
Genomic alteration landscape of each TME cluster. **(A)** The log10TMB of each cluster, gene-level CNV mutational signature, waterfall chart of significant (>5%) and differently (Fisher’s exact test p < 0.1) mutated genes, and heatmap of gene copy number variation were shown from the top to the bottom panels. Patients were ordered by the combined contribution of APOBEC-related mutational signatures (SBS2 + SBS13) with each cluster. **(B)** GISTIC copy number variation analysis. The amplifications and deletions of chromosomal regions were colored red and blue, respectively (FDR cutoff: 0.01).

To investigate the genomic alteration landscape of the TME cluster, we found that C3 showed a significantly higher tumor mutation burden and copy number variation than C1 (**Figure 5A**). By comparing genes with mutation rates greater than 5%, we observed that TP53, CTNNB1, OBSCN, DNAH7, CSMD1, RB1, FRAS1, and KMT2D, the most frequent alterations identified in LIHC (**Supplemental Table 3**), had significantly different patterns between clusters. Considering that the APOBEC family members, including APOBEC3A and APOBEC3B, catalyze mutation and promote cancer growth (26, 27), we chose the APOBEC activity-related mutational signatures, SBS2 and SBS13, to investigate the differences of the immune microenvironment among clusters. We found that C3 had more mutations in APOBEC-related signatures.

Through the description of the entire cluster of CNV landscapes (**Figure 5B**) by GISTIC 2.0, we discovered that the CNV of C1 was significantly less than those of the other three clusters. Although the degree of amplification was generally higher in C2, there were more amplifications and deletions of the chromosomal locus in C3. The chromosomal specificity loci, such as 11q13.3 and 9p21.3, only had no significant amplification in C1 (**Supplemental Tables 4**-**5**).

### Internal Validation and External Exploration of TME Clusters

**Figure 6:**
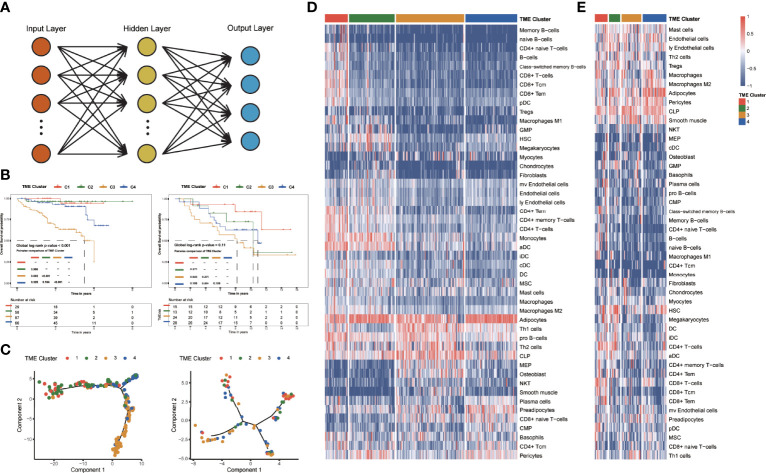
Identification and clinical characteristics of TME clusters in the verification group. **(A)** Neural network. The cell matrix was the input layer, and the subtype result was the output layer. **(B)** Kaplan–Meier curves for overall survival of 240 patients in the Japanese cohort (global log-rank test, p < 0.0001), and 80 patients in the Hoshida Y et al. cohort (global log-rank test, p = 0.11). **(C)** Pseudotime trajectory analysis of the Japanese cohort and the Hoshida Y et al. cohort. **(D)** Unsupervised clustering of TME cells for 240 patients in the Japanese cohort. **(E)** Unsupervised clustering of TME cells for 80 patients in the Hoshida Y et al. cohort.

The LIHC patients in TCGA dataset were randomly divided into the training cohort (n = 259) and the testing cohort (n = 111). We incorporated the matrix of putative cell scores into the neural network model and verified it in the validation set to determine TME clusters (**Figure 6A**). For internal validation, the accuracy of the neural network model was 0.949 in the testing cohort. In the external validation dataset, we used the neural network model to obtain the TME clusters and observed consistent survival differences between predicted TME clusters in the Japanese cohort and the Hoshida Y et al. cohort with that in TCGA dataset (**Figure 6B**).

To verify the stable interrelationship among the four subtypes, we performed pseudotime ordering analyses in the validation set with the same genes (**Figure 6C**). In all datasets, the patients in C1 and C3 were distributed at both endpoints of the hypothetical timeline and the patients of C2 and C4 were in the middle of the hypothetical timeline.

The landscape of infiltrating cells in TME was also explored (**Figures 6D, E**). Higher CD8+ T cell scores of C1 patients were observed in different validation datasets. Moreover, endothelial cells’ legible low-infiltrating score could be observed in C3 patients, and the Th 1 cells had a low infiltrating score in C4. These findings in validation datasets indicate a similar pattern of infiltrating cell enrichment with that of TCGA dataset.

### Therapeutic Response to Chemotherapy and Immunotherapy of Patients in Different TME Clusters

**Figure 7:**
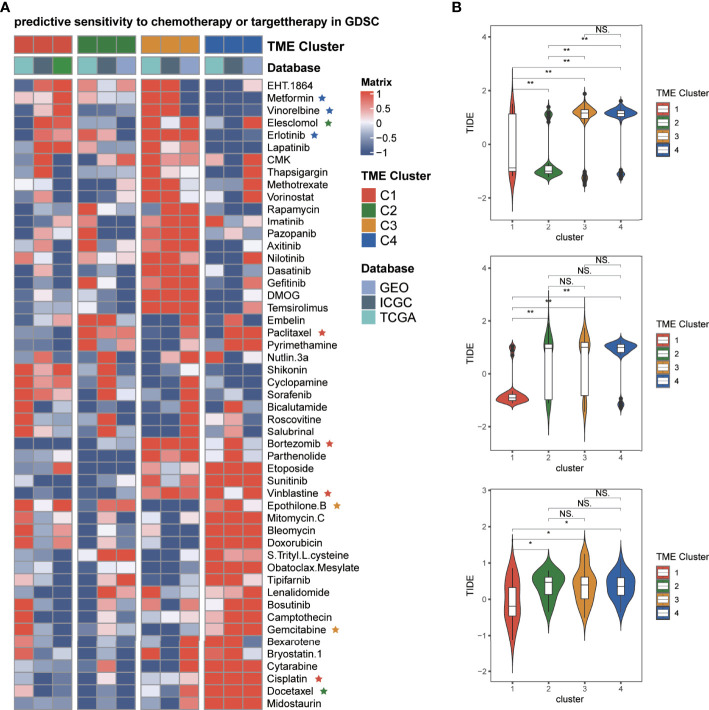
Predicting the efficacy of each TME cluster. **(A)** Heatmap of drug sensitivity in patients of different TME clusters in TCGA, Japanese, and Hoshida Y et al. cohorts. **(B)** Violin plots showed the comparison of the TIDE score of TME clusters in TCGA, respectively. *p≤ 0.05, **p≤ 0.01, ns: p> 0.05.

To find the response of LIHC patients to drugs in different TME clusters, we inferred the IC50 value of the 51 drugs in TCGA-LIHC, the Japanese cohort, and the Hoshida Y et al. cohort patients (**Figure 7A**). We found that patients in C1 might be more sensitive to paclitaxel, cisplatin, bortezomib, etc. The patients in C2 might be more sensitive to docetaxel, elesclomol, etc. The patients in C3 were less sensitive to most targeted drugs, except epothilone B and gemcitabine. Furthermore, patients in C4 might be more sensitive to metformin, vinorelbine, erlotinib, etc. The potential response of ICB therapy was estimated by the TIDE algorithm, which can evaluate the efficacy of anti-PD1 and anti-CTLA4 treatments. We discovered that C1 had the lowest TIDE score than other clusters (**Figure 7B**), which means the patients of C1 may get more benefits from immunotherapy. Comparing the scores of C2 patients in three cohorts, we speculated that C2 patients could not benefit from immunotherapy stably. Moreover, the patients of C3 and C4 may have poor immunotherapy efficacy.

## Discussion

We identified four tumor microenvironment subtypes based on the lymphocytes, myeloid cells, stem, and stromal cells and discussed the difference of survival, cell infiltration, tumor mutation burden, copy number variation, and functional enrichment pathways between the clusters. A neural network model was established based on the obtained cell matrix and verified in the Japanese and Hoshida Y et al. cohorts. In conclusion, the multiple signatures suggested that the long-term outcome and immunotherapy efficacy of patients may be different among four TME subtypes.

It was generally considered that the hematopoiesis becomes skewed toward myeloid and away from lymphoid lineages with age; therefore, aging negatively impacted CD8+ T cell immunity and positively connected with adipocytes (28). Similar trends may also exist in the tumor microenvironment of LIHC patients. The increase of adipocytes and the decrease of granulocyte-macrophage progenitor may indicate the transformation to the aging microenvironment, which may dramatically affect tumor progression (29). Our study suggested that macrophage M2 was increased in older LIHC patients, with obvious tumor-promoting functions (30). Tumor-associated adipocytes can promote the immunosuppressive TME and aggravate tumor progression (31). Considering the immune cell-adipocyte cross talk mentioned in single-cell studies, the growth of adipocytes may cause systemic energy imbalance in TME (32). In summary, we speculated that elderly patients might have a more immunosuppressed TME than young patients.

Besides, most of the previous research was based on the immune microenvironment and did not analyze the types of stromal cells and stem cells. Our research explored immune and non-immune cells in the immune microenvironment of liver cancer, which makes our study more complete and closer to the real situation. The TME component of LIHC may be unique, such as the existence of endothelial cells. Highly endothelial cell infiltration was usually associated with poor survival, and the liver sinusoidal endothelial cells could not be ignored in the liver, which has vital physiological and immunological functions, including antigen presentation, and leukocyte recruitment (33). In liver cancer, a high endothelial cell score is associated with a good prognosis, and correspondingly, the patients of C3 have a lower endothelial cell score and worse prognosis. Likewise, in the non-neoplastic cirrhotic and non-cirrhotic liver, PD-L1 was expressed on Kupffer cells (macrophages that reside in hepatic sinusoids), endothelial cells, and immune cells, all of which scored lowly in C3 (34). This result is consistent with the low expression of CD274 in C3, which also indicates that immunotherapy may be less effective in C3. In addition, a single-cell sequencing study found that exhausted CD8+ T cells and regulatory T cells were preferentially enriched and potentially clonally expanded in LIHC, but the functions of CD8+ T cells were repressed (35). Although the patients of C2 had a higher CD8+ T cell score than C1, the functions of CD8+ T cells may be inhibited.

We observed that the enrichment of pathways in different types seems regular. For instance, the cell cycle and cell proliferation-related pathways had the highest enrichment in TME C3, followed by C2, C4, and C1. In immune-related pathways, the enrichment of C1 and C2 was opposite to that of C3 and C4. These results indicated that the immune phenotype of C1 may be similar to that of C2 and antagonistic to that of C3 and C4. Simultaneously, the cell proliferation-related pathways in C1 that had the lowest enrichment may indicate that the cancer cells were less malignant, which explained the better survival outcome of patients in C1. Interestingly, the patients of C1 had a relatively inactive immune microenvironment compared with patients of C2. The immune cell scores, stromal fraction, and immune pathway enrichment were similar in C1 and C2. Intriguingly, the expression of immune-related genes of C2 was even higher, but C2 did not have a better survival outcome than C1. Regarding the metabolism laws of the four subtypes, we believed that the metabolism and cell cycle of C1 were relatively inactive, and there is an evolutionary process from C1 to high-metabolome subtypes by pseudotime ordering analysis. Considering that the enrichment of immune and metabolic-related pathways in C2 was higher than that in C1, we speculated the high immune activity could not control more malignant tumors in C2, which may explain why the survival outcome of C2 was worse than that of C1. In addition, the TIDE score consisted of a dysfunction score and an exclusion score, which were associated with the average expressions of CD8A, CD8B, GZMA, GZMB, and PRF1 (20). The above cytotoxic T lymphocyte markers were expressed differently between C1and C2; therefore, the TIDE scores between C1 and C2 were different.

As for the differences of TMB in each cluster, proliferative hepatocellular carcinoma was associated with chromosomal instability and TP53 mutations, and non-proliferative tumors were a well-differentiated phenotype with CTNNB1 mutations (36). The TP53 mutation frequency was higher in C2; we speculated that the patients of C2 may belong to the proliferative hepatocellular carcinoma. Involved in CNV, although the amplified chromosomes were at the same locus, the amplified genes were not the same among clusters. Oncogenes expressed by chromosomal 11q13.3, such as CCND1 and some FGF family genes, increased the immune checkpoint signature expression, including CD274, PDCD1, BTLA, CTLA4, HAVCR2, IDO1, and LAG3 (37). We discovered that CCND1 was only amplified in C2, which may account for the high expression of immune checkpoint signatures in C2. There have been targeted drugs applicated for 11q13.3 amplification (38). However, the amplified genes were significantly different between clusters, and only patients in C3 were accompanied by amplified FGF family genes, which suggested a better response to FGF inhibitors. It is also noteworthy that FGF3 amplification may be associated with multiple lung metastases and a poorly differentiated tumor (39).

We also observed a similar situation at site 9p21.3. LIHC does not belong to the frequent 9p21 loss cancer type, but this CNV type can distinguish between subtypes with obvious deletion (C1) and no apparent deletion (C2–4), according to our study. The 9p21 loss correlates with “cold” tumor-immune phenotypes and shorter survival (40). In melanoma, the deletion of 9p21 was associated with primary resistance to anti-PD-1/PD-L1 monotherapy, suggesting that immunotherapy may not be effective in C2–4. On the other hand, we must pay attention to the high expression of CD274 in C2, suggesting that C2 patients may benefit from immunotherapy. Combined with 9p21.3 loss, the immunotherapy efficacy in these patients may be variational. Moreover, this conclusion is the same as the result calculated by TIDE in our study. In parallel, patients with deletions or mutations in CDKN2A and CDKN2B, common deletion genes in C2–4, had a significantly shorter time to progress on chemotherapy (41). In contrast, C2 and C3–4 have different genes deleted at the chromosomal 9p21. The patients of C2 had more CDKN2B and CDKN2B-AS1 deletions than C3–4. The CDKN2B-AS1 knockdown inhibited cell proliferation, migration, and invasion and induced G1 arrest and apoptosis of tumor cells (42). In short, C2 patients may have fewer metastases than C3 and 4.

In brief, the patients of C1 have lower malignancy and higher immunological activity, without 9p21.3 deletion or 11q13.3 amplification, and can achieve better curative effects in immunotherapy. C2 patients have high immunological activity and a high expression of immune checkpoint inhibitors. The malignance of the two clusters may be lower and tumor not easy to metastasize, and both of them may benefit from immunotherapy or local therapy. The patients of C3 with lower immune and stromal cell infiltration, and highest tumor purity, find it challenging to benefit from immunotherapy. They may get better curative effects from drugs that target FGF/FGFR, including lenvatinib (43). The patients of C4 may belong to immune-insensitive subtypes like C3 and develop toward C3. Considering that the cell-division M phase-related pathways, like the sister chromatid segregation pathway, were highly enriched in C3, the vinorelbine drugs may achieve good results in C4.

Although our study confirmed four TME subtypes that potentially predicted antitumor treatment efficacy, our research still has limitations. The experimental data were lacking in our study, and the evaluation of efficacy of therapies mentioned in our study should be performed in larger-scale clinical data. In conclusion, our study laid an accurate foundation of four TME subtypes, which may provide therapeutic inspiration for patient selection for appropriate therapies in LIHC.

## Data Availability Statement

Publicly available datasets were analyzed in this study. These data can be found here: https://dcc.icgc.org https://www.ncbi.nlm.nih.gov/geo/query/acc.cgi?acc=GSE10141.

## Author Contributions

Research design: JW, HZ, BX, and YY. Data collection: CZ, JH, and JZ. Data analysis: HZ. Manuscript preparation: HZ and JW. Chart preparation: HZ and JW. Revisions: YY, JW, BX, and HZ. All authors confirm that they contributed to the manuscript review and critical revision for important intellectual content and read and approved the final draft for submission. All authors are also responsible for the manuscript content.

## Funding

This work was supported by grants from the Wuhan University Medical Faculty Innovation Seed Fund Cultivation Project (grant no. TFZZ2018025), Chen Xiao-Ping Foundation for the Development of Science and Technology of Hubei Province (grant no.CXPJJH12000001-2020313), the National Natural Science Foundation of China (grant nos. 81670123 and 81670144), the Fundamental Research Funds for the Central Universities (grant no. 2042021kf0129), the Fundamental Research Funds for the Central Universities (grant no. 2042021kf0129), and the Nature Science Foundation of Hubei Province (grant no. 2021CFB086).

## Conflict of Interest

The authors declare that the research was conducted in the absence of any commercial or financial relationships that could be construed as a potential conflict of interest.

## Publisher’s Note

All claims expressed in this article are solely those of the authors and do not necessarily represent those of their affiliated organizations, or those of the publisher, the editors and the reviewers. Any product that may be evaluated in this article, or claim that may be made by its manufacturer, is not guaranteed or endorsed by the publisher.
