# Supplementary material for: Comprehensive Analysis Identifies and Validates the Tumor Microenvironment Subtypes to Predict Anti-Tumor Therapy Efficacy in Hepatocellular Carcinoma
Source: Front Immunol. 2022 Jul 18;13:838374. doi: 10.3389/fimmu.2022.838374 (PMC9339643; doi:10.3389/fimmu.2022.838374)
Supplement: Supplementary file 1 [file DataSheet_1.docx]

Supplementary Material

# Supplementary Figures and Tables

## Supplementary Figures

**
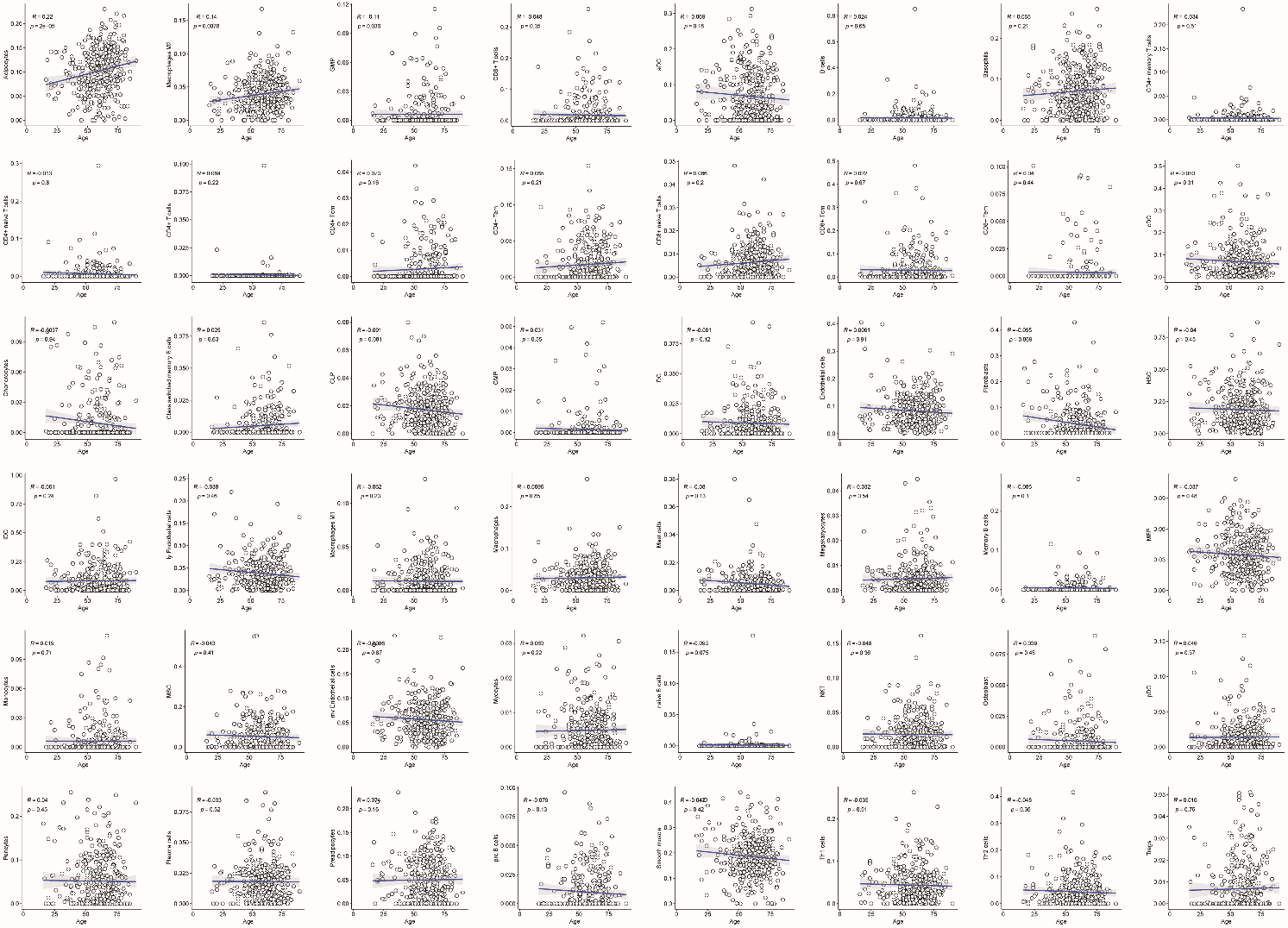
**

**Supplementary Figure 1.** The associations between age and each cell score in TME of patients.


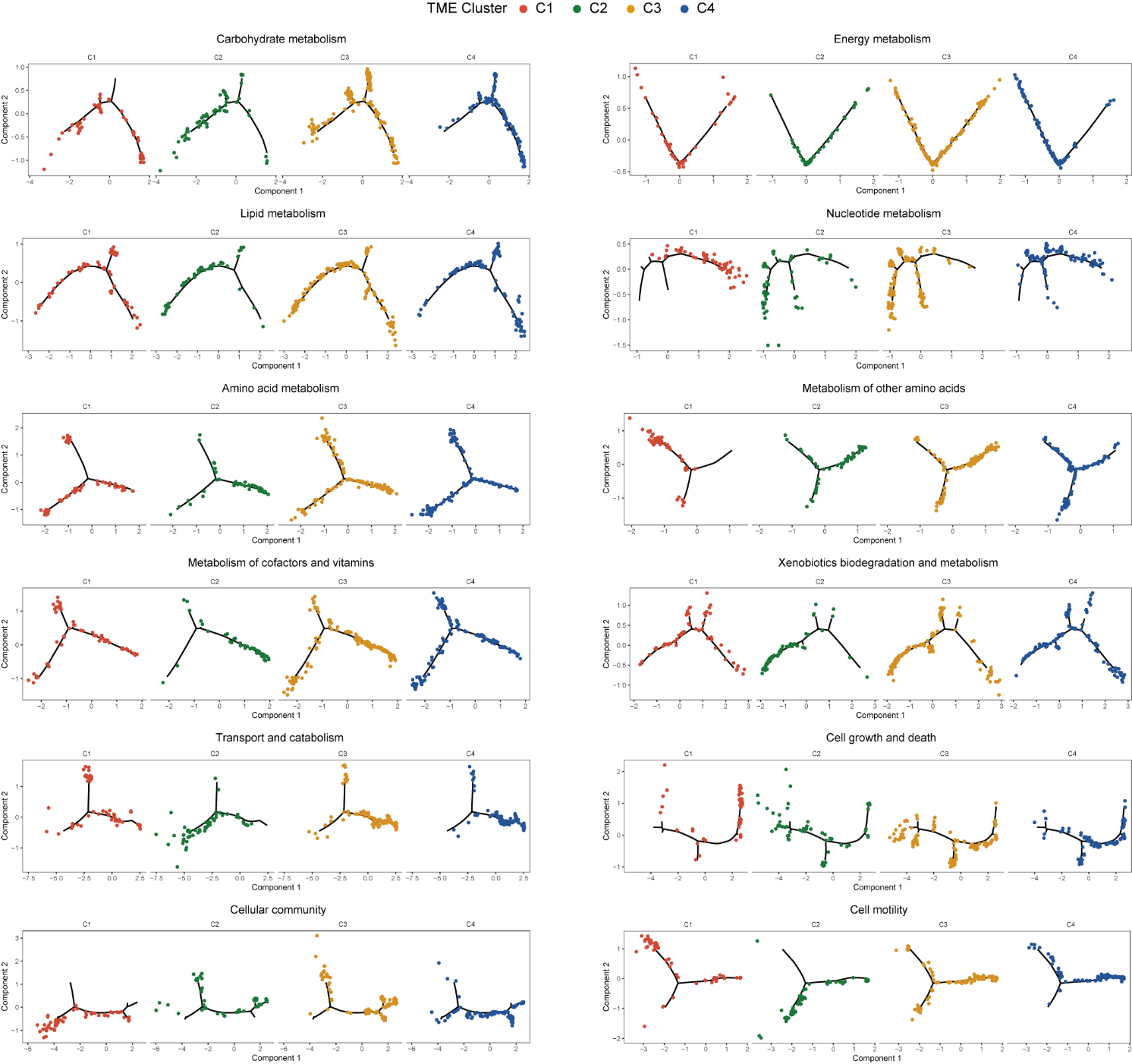


**Supplementary Figure 2.** Pseudotime trajectory analysis of 370 patients in the TCGA based on the carbohydrate metabolism, energy metabolism, lipid metabolism, nucleotide metabolism, amino acid metabolism, metabolism of other amino acids, metabolism of cofactors and vitamins, xenobiotics biodegradation and metabolism, transport and catabolism, cell growth and death, cellular community, and cell motility pathways related genes.

**
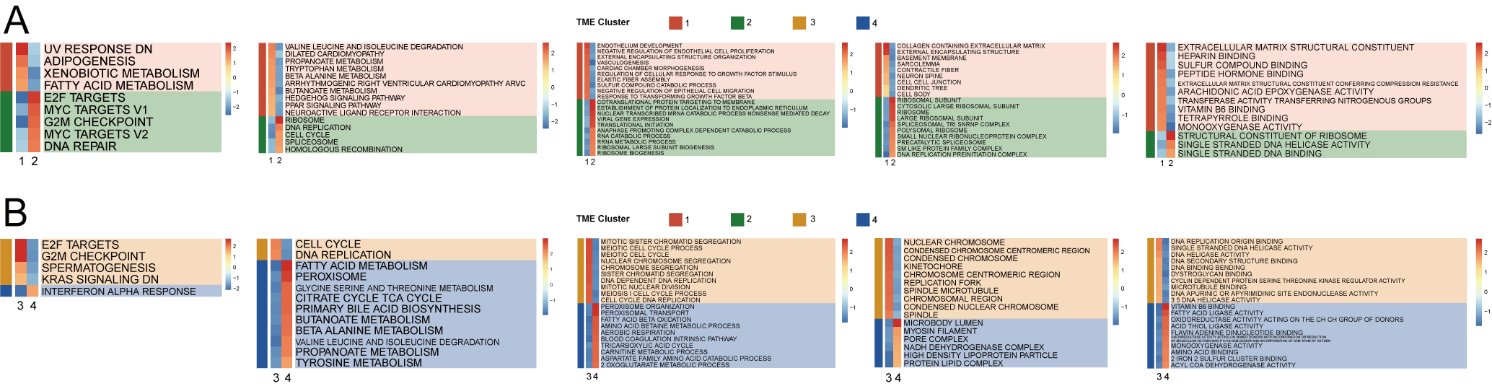
**

**Supplementary Figure 3.** Comparison of enrichment pathways between similar clusters. (A) Comparison of differential enrichment pathways between C1 and C2 (The normalized enrichment score (NES) > 1; P < 0.05 versus NES < -1; P < 0.05). (A) Comparison of differential enrichment pathways between C3 and C4 (The normalized enrichment score (NES) > 1; P < 0.05 versus NES < -1; P < 0.05).

## Supplementary Tables

**Supplementary Table 1.** The univariable Cox analysis of 48 cells and overall survival.

**Supplementary Table 2.** Differentially expressed genes of each cluster.

**Supplementary Table 3.** Fisher test for significant mutated genes in each cluster.

**Supplementary Table 4.** Amplification genes located within chromosome locus of each cluster.

**Supplementary Table 5.** Deletion genes located within chromosome locus of each cluster.
